# Hematopoietic Stem Cell Metabolism during Stress and Aging

**DOI:** 10.20900/agmr20260018

**Published:** 2026-07-09

**Authors:** Devyani Sharma, Marie-Dominique Filippi

**Affiliations:** 1 Division of Experimental Hematology and Cancer Biology, Cincinnati Children’s Hospital Research Foundation, Cincinnati, OH 45229, USA; 2 Cancer and Cell Biology graduate program, University of Cincinnati College of Medicine, Cincinnati, OH 45229, USA

**Keywords:** hematopoietic stem cells, HSC aging, metabolic remodeling, mitochondrial metabolism, stem cell quiescence, oxidative phosphorylation, fatty acid oxidation, bone marrow niche, stress hematopoiesis, mitochondrial quality control

## Abstract

Hematopoietic stem cells (HSCs) support the lifelong production of blood but undergo substantial changes during aging. Aging causes a progressive decline in the repopulation ability of HSC with changes in the proportion of myeloid and lymphoid lineages. Metabolic remodeling is increasingly recognized not only as a consequence of HSC aging but also as an active contributor to age-associated changes in HSC function. Quiescent HSCs have low metabolic activity, in contrast with their highly proliferative progeny. During regeneration, drastic metabolic remodeling enables HSC to exit quiescent and proliferate to sustain blood production for emergency hematopoiesis. Metabolic activity drifts during aging. Changes in glycolysis, mitochondrial activity, and lysosomal functions all contribute to a slow decline of the hematopoietic system. In this review, we focus on the metabolic needs of HSCs, how they control their quiescence and proliferation, and how a metabolic drift contributes to HSC aging.

## INTRODUCTION

Aging is associated with chronic diseases due to a progressive loss of tissue integrity. In the hematopoietic system, aging leads to deterioration of the adaptive immune system [1], but overproduction of myeloid cells anemia, causing a state of immunodeficiency and chronic inflammation. Aging increases incidence of clonal hematopoiesis (i.e., expansion of one HSC clone carrying acquired mutations), myeloid proliferative diseases and hematopoietic neoplasms [2,3]. Aging arises from the progressive decline of hematopoietic stem cell (HSC) functions. HSCs are rare cells that reside in the bone marrow of adult mammals [4]. They are functionally characterized by their ability to reconstitute the entire blood components of an organism [5]. At the same time, HSC can self-renew to maintain a functional HSC pool throughout life. Since mature blood cells are short lived, HSCs are required to undergo division to consistently produce multilineage progenitors and committed single lineage precursors. These divisions ultimately result in continuous production of cells of all lineages leading to formation of different components of blood, but also HSC functional decline and loss of plasticity [6]. During stress, certain cytokines including interferon α [7], interferon γ [8], and granulocyte colony stimulating factor (G-CSF) [9] trigger HSC quiescence exit to meet increased demands during emergency situations such as blood loss or infections. These properties are largely altered with aging, and aging HSCs accumulate cellular damage that impair cellular functions [10]. Extensive literature has reported that aged HSCs (18–24 months old) are phenotypically and functionally impaired. Aged HSCs accumulate in number in the bone marrow of older mice but are characterized by their low regenerative activity [11–14] and by a tendency to produce more myeloid cells at the expense of lymphoid lineages [15].

Metabolic activity has emerged as a critical regulator of HSC functions, and control both HSC quiescence and activation into cycle. Exit from quiescence and subsequent differentiation is integrated with metabolic remodeling to meet the demand [16]. HSCs are known to depend on anaerobic glycolysis and exhibit low metabolic and biosynthetic activity in order to maintain quiescence for long period of time and be protected from oxidative stress [17–23]. On the other hand, higher mitochondrial activity is commonly seen during HSC commitment to differentiation, and their immediate progeny. They exhibit higher metabolic activity and increased oxidative phosphorylation [16] to quickly enter the cell cycle when needed to meet the hematopoietic demand [24,25]. Metabolic programs are tightly regulated to meet the cellular demand. Metabolic regulation progressively declines with aging, and it has become clear that it is not a consequence but rather a determinant of aging. This review intends to summarize the recent advances in our understanding of the metabolic control of HSC functions, what we know about metabolic deregulation with age and how it drives the aging functional decline.

## HSC QUIESCENCE AND METABOLIC CHECKPOINT

### Hypoxic Niche and Glycolysis

HSC self-renewal and differentiation is regulated by the BM microenvironment, or ‘niche’, in which they are residing [26]. The niche controls access to oxygen and nutrients and provides signaling cues that can shift metabolic state and influence whether HSCs remain quiescent or become activated. Changes in niche composition, cytokines and inflammatory signaling can alter the balance between quiescence and activation and may contribute to long-term loss of HSC function. The microenvironment is heterogeneous with peri-arteriolar and peri-sinusoidal regions that contain different oxygen levels [27–29]. HSC reside in low oxygen-tension microenvironment, named “hypoxic niche”. In the hypoxic environment, quiescent HSCs rely on anaerobic glycolysis rather than mitochondrial oxidative phosphorylation, to meet their energy demands and maintain their functions [21–31]. HSCs maintain intracellular hypoxia via tightly controlled hypoxia-linked programs (*Hif1a*), which enables [32] HSC to use anaerobic glycolysis and bypass [33]. mitochondrial respiration. There is ample evidence indicating that maintaining anaerobic glycolysis is critical to maintain HSC quiescence. When HIF1A or pyruvate dehydrogenase (*Pdk2* and *4*) are depleted, HSCs exhibit increased mitochondrial metabolism, lose their quiescent state and eventually functionally exhaust [33,34]. This glycolytic phenotype is also regulated by the transcription factor MEIS1 that preserves the metabolic phenotype and oxidant defense of HSCs [35].

Although glucose is an important nutrient for quiescent HSCs, it must be controlled. In fact, enhanced glycolysis promotes HSC cycling. It has been shown that the most deeply quiescent HSCs show less glycolytic dependency for their survival and functions than proliferating progeny. [36] This is important because it argues that the metabolic program that maintains quiescent HSCs is more complex. The deepest quiescent state seems to be associated with a restrained metabolic program rather than just glycolysis. This suggests that enhanced glycolysis marks a primed state closer to activation, whereas deep quiescence depends on tight nutrient control.

Other nutrients are also linked to HSC quiescence, such as vitamin A-retinoic acid signaling [37,38]. Retinoic Acid (RA)-induced signaling is highly enriched in dormant label-retaining HSCs [38]. Interestingly, in the presence of the RA signaling agonist all-trans retinoic acid (ATRA), HSCs were significantly more quiescent with lower nascent protein synthesis and lower mitochondrial activity [37]. Subsequent studies showed that retinoic acid signaling controls HSC quiescence via its downstream product 4-oxo-RA under the control of the cytochrome P450 family 26 subfamily B member 1 (*Cyp26b1*), which is highly abundant in HSCs [39]. Ascorbate (vitamin C) is another vitamin found highly enriched in HSCs and progenitors, although this may limit HSC functions [40]. The exact physiological importance of this pathway and how it is integrated with other metabolic activity of HSC remains to be investigated.

### Restricting Mitochondrial Output Matters to Maintain HSC Quiescence

While the glycolytic program shapes the steady-state metabolic state of HSCs, HSCs contain high mitochondrial content and must restrict mitochondrial activity to preserve long-term stem cell function. Studies using the mitochondrial reporter mouse expressing the Dendra2 fluorescence onto mitochondria show that HSCs can be separated into mito-Dendra2^Low^ and mito-Dendra2^High^ subsets [41], representing HSCs with low and high mitochondrial content, respectively. Interestingly, mito-Dendra2^High^ HSCs exhibited deeper quiescence but higher regenerative capacity in transplant studies than mito-Dendra2^Low^ HSCs [41]. Despite having high mitochondrial content, mitochondrial activity is reduced in HSCs. Numerous studies have now shown that the long-term repopulation activity of HSCs is found in the TMRE low fraction of phenotypically defined HSCs, representing HSCs with lower mitochondrial membrane potential [21,23,36,42,43]. This is conserved in humans, in which the most potent human HSCs are enriched with the mitochondria that has the lowest membrane potential [44].

Restraining mitochondrial functions is important because increased mitochondrial activity raises reactive oxygen species (ROS) levels, which has negative impacts on HSC functions. Previous work showed that excessive ROS activate stress pathways such as p38^MAPK^ and limit HSC lifespan, indicating that redox balance is critical to maintaining self-renewal capacity [45,46]. During steady state, this is prevented by mechanisms that keep mitochondrial biogenesis and oxidative metabolism under restraint. Work from Chen et. al. showed that the TSC-mTOR pathway maintains HSC quiescence and function by limiting mitochondrial biogenesis and ROS production, and loss of this control drives HSCs out of quiescence and into rapid cycling [47]. In parallel, FOXO-dependent antioxidant programs protect HSCs from physiological oxidative stress and preserve their long-term functions [46,48]. Consistently, HSCs have abundant lysosomes but with low degradative activity, and restraining lysosome activity maintains HSC quiescence and potency [36,49]. Together, these findings show that restricting mitochondrial output is not simply a way to lower energy production, but a key part of the steady-state checkpoint that limits oxidative damage, preserves quiescence, and protects the long-term integrity of the HSC pool.

### Mitochondria Are Not Completely Silent in HSCs

**Figure 1.**
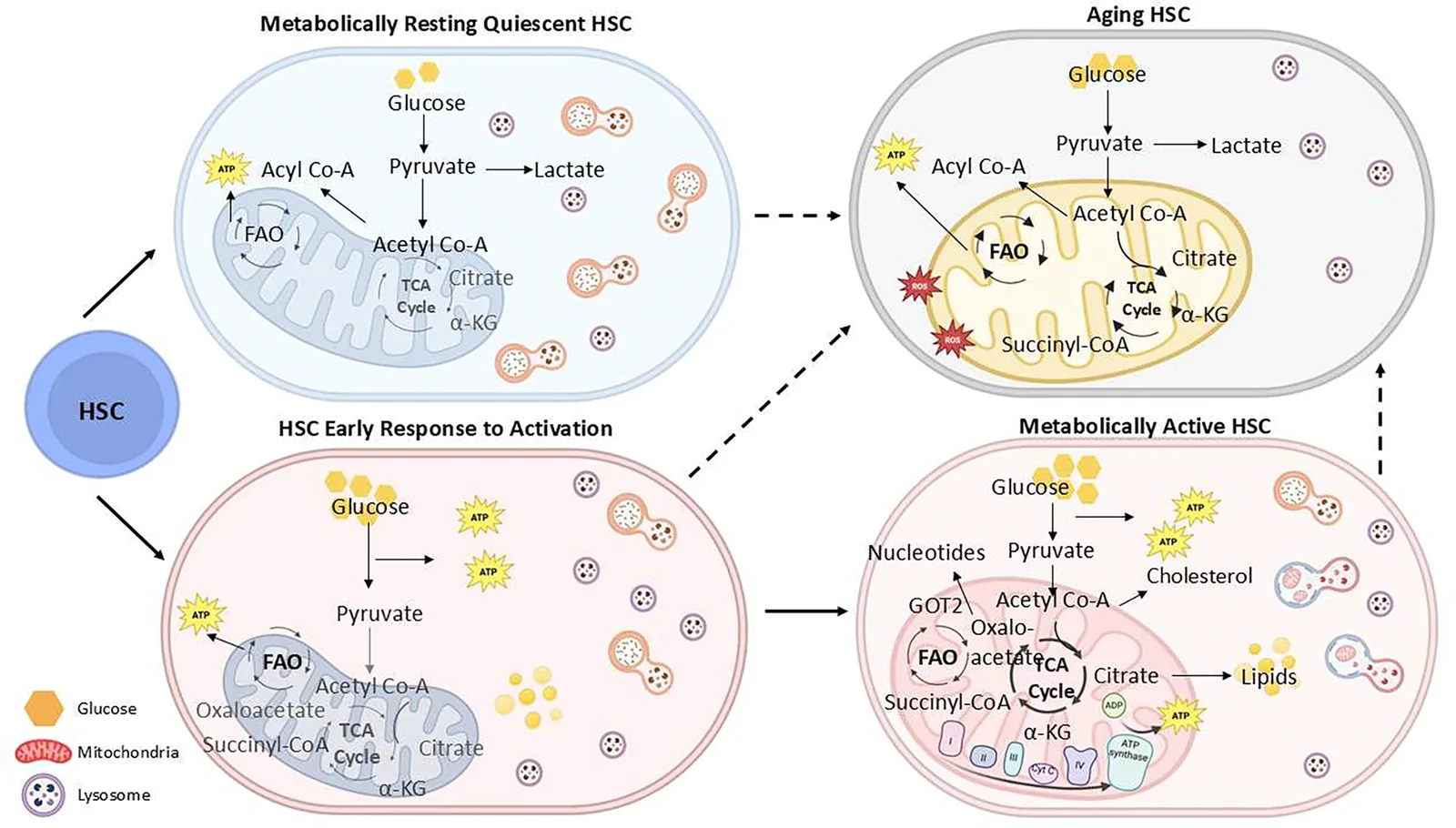
Metabolic plasticity between resting and active HSC state. In the resting state, HSC metabolic activity is restrained, and energy production is lower which is consistent with reduced cellular demand. Upon activation, HSC mitochondria show increased respiratory function and ATP generation to meet the high energy demand. Aging is associated with metabolic drifts and adaptations.

Interestingly, mitochondrial metabolism remains important to maintain HSC functions. Fatty acid oxidation (FAO) in mitochondria is important to maintain HSC functions [50,51]. Fatty acids (FA) are transferred into mitochondria via the mitochondrial carnitine palmitoyl transferase I (*Cpt1a*) where they are broken down into acetyl-CoA, known as beta-oxidation. Acetyl-CoA generated from FAO can fuel the tricarboxylic acid (TCA) cycle for energy production or be used in the cytoplasm for de novo lipid synthesis. FAO is active in HSCs and is higher than in more differentiated bone marrow cells [50,52–54]. HSCs can rely on FAO to maintain their quiescence and self-renewal activity [52]. It remains unclear how FAO is used in HSCs, and other metabolic pathways are likely important as well, some studies but not all have found that loss of *Cpt1a* alters HSC regenerative potential in transplant studies [53,54] (Figure 1 and Table S1).

### HSC Quiescence and Mitochondrial Quality Control

Mitochondrial quality control is important to maintain HSC functions [55]. Mitochondria have high turnover rate and are highly dynamic. Like other cells, HSCs need tight mitochondrial quality control mechanisms, including autophagy and mitophagy to remove damaged organelles, and fusion/fission cycles to remodel the mitochondrial network. Loss of the autophagy regulator Atg7 results in accumulation of mitochondria in HSCs associated with higher ROS levels, increased HSC proliferation and loss of normal HSC function [55]. Further, deletion of *Atg12* in adult HSCs impaired self-renewal and caused myeloid-biased output. Autophagy-deficient HSCs accumulated elongated and fused mitochondria that had higher mitochondrial membrane potential, increased oxidative metabolism, higher ROS, increased protein synthesis, and more cycling activity. These changes closely resembled features of old HSCs, suggesting that autophagy is needed not only for organelle turnover but also to keep HSC metabolism restrained and preserve stemness over time [56]. Interestingly, Chua et al. used an autophagy reporter model to show that HSC repopulation activity is found in the HSC fraction that has high autophagy activity [57], perhaps consistent with high lysosome content in HSC [36]. The organization of the mitochondrial network is plastic and varies with HSC subtype. Lymphoid-biased HSCs express higher levels of fusion regulator *Mfn2* than myeloid-bias HSCs, and their mitochondria are more elongated in shape [58]. Interestingly, MFN2 is important to maintain quiescence of lymphoid-bias HSCs by controlling the tethering mitochondria to the endoplasmic reticulum to enhance intracellular calcium buffering [58]. Together, steady-state HSC quiescence is supported by an active metabolic program that favors glycolysis, limits mitochondrial respiration and ROS, and protects long-term stem cell integrity. Steady-state HSCs are metabolically restrained but still preserve enough mitochondrial fitness to remain functional.

## AGING HSCS AND THE DRIFT IN METABOLIC ACTIVITY

### Mitochondrial Functions in Aging HSCs

Aged HSCs are accompanied by clear changes in their mitochondrial state. Aged HSCs have higher mitochondrial mass but lower mitochondrial membrane potential than younger HSCs [56,59–61]. The mitochondrial network is more globular and fragmented, and it tends to be polarized [61]. Interestingly, not all aged HSC have mitochondrial defects. Aged HSCs, like young HSCs, can be separated into functionally distinct HSC populations based on their mitochondrial mass and membrane potential state. Like young HSCs, repopulation activity was found in the aged HSC subset with the highest mitochondrial mass [59]. The repopulation activity was also found in the aged HSC subset that retains mitochondrial potential like young HSCs. This aged HSC subset retained more transcriptional features of young HSCs, with more balanced lineage programs [60]. Old HSCs with decreased mitochondrial membrane potential showed more typical aged phenotype with reduced fitness and myeloid bias, indicating that loss of mitochondrial membrane potential is a cause of functional defects, but mitochondrial accumulation helps maintaining HSC functions [60]. This results in metabolic drift. Aged HSCs have higher OXPHOS activity. Aged HSCs are less glycolytic and become more dependent on FAO than young HSCs [51,54], perhaps due to the chronic inflammation environment. Inflammation seems to impair glucose uptake and suppress glycolysis in aged HSCs through Socs3-mediated inhibition of AKT/FOXO-dependent signaling [51]. A recent study from the Morrison group showed that long-chain FAO becomes necessary during aging [54]. The same study also showed that a high-fat diet increases FAO and reduces HSC function, indicating that increased FAO is not always beneficial [54] (Figure 1 and Table S1).

### Aging and Loss of Mitochondrial Quality Control Mechanisms

Aged HSCs accumulate dysfunctional mitochondria and metabolic activity due to defects in mitochondrial quality control, including reduced autophagy and mitophagy [56,59]. Aged HSCs have reduced mitochondrial unfolded protein response due to decreased *Sirt7* expression and causing increased mitochondrial activity [10]. Lysosomal dysfunction is also a major feature of aged HSCs. Aged HSCs have fewer but more acidic lysosomes than young HSCs. Lysosomes are also damaged and abnormally activated. Lysosomal dysfunction caused impaired processing of mitochondrial DNA leading to increased activation of the DNA sensing machinery, cGAS-STING, and subsequent interferon signaling, further supporting the idea that aging HSCs lose tight coordination between organelle quality control, mitochondrial homeostasis, and stress signaling [62]. Interestingly, suppressing lysosomal hyperactivation was sufficient to restore lysosomal integrity along with metabolic and epigenetic homeostasis [62]. This suggests that in young HSCs, mitochondrial activity and organelle quality are held in a tight range. In old HSCs, this balance becomes altered, which affects quiescence, regenerative capacity, and lineage output.

### Metabolic Resilience

Despite accumulating abnormal mitochondria, and having higher ROS levels, recent work suggests that aged HSCs survive and acquire metabolic resilience through changes in metabolic dependencies. Watanuki et al. (2024) [63] showed that old HSCs are metabolically reprogrammed in a way that helps them survive stress. They showed that at steady state, aged HSCs activate the pentose phosphate pathway, making them more resistant to oxidative stress and less dependent on glycolytic ATP production. Under metabolic stress, aged HSCs can also rapidly increase mitochondrial ATP production through enhanced complex II metabolism. This response depends on increased SDHAF1, a factor required for succinate dehydrogenase assembly. This program is supported by physiologically low concentration thrombopoietin exposure in the bone marrow environment. This suggests that some aged HSCs may not only show metabolic decline but may be capable of activating adaptive pathways that confer survival advantage despite overall functional decline. Consistently, a subset of aged HSCs tends to maintain normal functions notably by maintaining autophagy as an adaptive cytoprotective response to chronic inflammation [51]. Hence, aged HSCs survive with abnormal mitochondria but adapt and shift metabolic dependencies [60,64].

## ACUTE STRESS HEMATOPOIESIS

Acute stress hematopoiesis refers to the demand-driven increase in blood cell production that occurs when the hematopoietic system is challenged. Events such as infection, inflammation, blood loss, cytotoxic injury, or other tissue damage can be considered as such challenges. In such scenarios, HSCs and progenitor cells are pushed out of steady-state behavior to increase proliferation and differentiation in order for the blood system to quickly restore immune and hematopoietic function [65]. Acute stress hematopoiesis forces HSCs out of quiescence, which requires a highly coordinated metabolic remodeling to meet the energy and biomass demand.

### Acute Stress and Exit from Quiescence

HSC activation is accompanied by metabolic remodeling. Numerous studies have shown that activated HSCs rapidly acquire higher mitochondrial membrane potential, increased OXPHOS and calcium signaling, which is necessary for cell cycle progression [56,66–68]. Interestingly, increased glycolysis is also important during HSC activation with increased expression of glycolytic genes [36,66]. Cycling-primed HSCs readily depend on glycolysis [36]. A recent study showed that under proliferative stress, HSCs rapidly increase anaerobic glycolysis through the glycolytic rate limiting enzyme PFKFB3. HSCs increase the activity of PFK during proliferation also when OXPHOS is inhibited, maintaining the cells need for their ATP production. This response is directly required to support stress hematopoiesis [63]. Thus, early stress activation is not simply a shift towards mitochondrial oxidation, but glycolysis plays a central role during the early stage of activation.

Mitochondrial activity still supports HSC proliferation and recovery. Ito. et al. showed that self-renewing expansion of Tie2^+^ HSCs, a minimally heterogenous murine HSC population, depends on peroxisome proliferator-activated receptor (PPAR), which is a fatty acid oxidation pathway, via enhanced Parkin recruitment on mitochondria. This was a connecting link between mitochondrial clearance and fatty acid oxidation to stem cell maintenance during proliferative conditions [50]. Mitochondrial respiration seems to be important as well. Expression of ETC proteins increases to form a fully functioning mitochondrial respiratory system [69]. Defects in mitochondrial respiration, via deletion of the mitochondrial complex III subunit Rieske iron sulfur protein (RISP) or PTEN-like mitochondrial phosphatase (*Ptpmt1*) reduced HSC quiescence resulting in bone marrow failure [70,71]. We just reported the importance of branched chain amino acid (BCAA) catabolism during HSC activation to maintain HSC self-renewal [43]. We found that HSCs with the highest repopulation potential exhibit high BCAA catabolism activity via the BCAA transaminase *Bcat2*, to keep the cell cycle length under control and self-renew. HSC regeneration potential decline when BCAA catabolism is inhibited and the BCAA are used for anabolic reactions instead [43]. This necessitates a highly dynamic mitochondrial network. We previously showed that the organization of the mitochondrial network changes every few minutes in activated HSCs in a manner dependent on the mitochondrial fission regulator *Drp1*; and this is needed for full HSC regenerative potential [68].

Other metabolic pathways are important to meet the need of proliferating cells in building biomass, including proteins, lipids and nucleotides. For instance, p38 mitogen-activated protein kinase activity increases during HSC activation, and promotes the expression of both inosine-5-monophosphate dehydrogenase 2 (*Impdh2*), the rate-limiting enzyme of guanosine monophosphate (GMP) synthesis, and guanosine monophosphate synthetase (*Gmps*), which is necessary for HSPC cycling [66]. Aspartate metabolism via glutamic-oxaloacetic transaminase 2 (*Got2*) in mitochondria is also important to fuel the TCA cycle during HSC proliferation for nucleotide synthesis [72]. The ATP citrate lyase (*Acly*) increased in HSCs in the early phase after the administration of 5-FU, along with greater glucose uptake and higher intracellular levels of glutamate [73]. Fatty acid desaturase 2 (*Fads2*), which catalyzes the rate limiting step in the desaturation of fatty acid is critical for regenerating the hematopoietic system upon myeloablative chemotherapy [74]. HSCs also need a tight control of cholesterol metabolism. HSCs with high cholesterol content tended to have higher regenerative functions [75]. During acute infection, Mistry et al. (2021) [76] showed that HSCs increase *CD36*-dependent free fatty acid uptake and shift toward FAO to support HSC activation and emergency hematopoiesis. How these pathways are fully integrated is not yet known but will be important to investigate (Figure 1 and Table S1).

### Return to Quiescence—An Active Metabolic Reset

**Figure 2.**
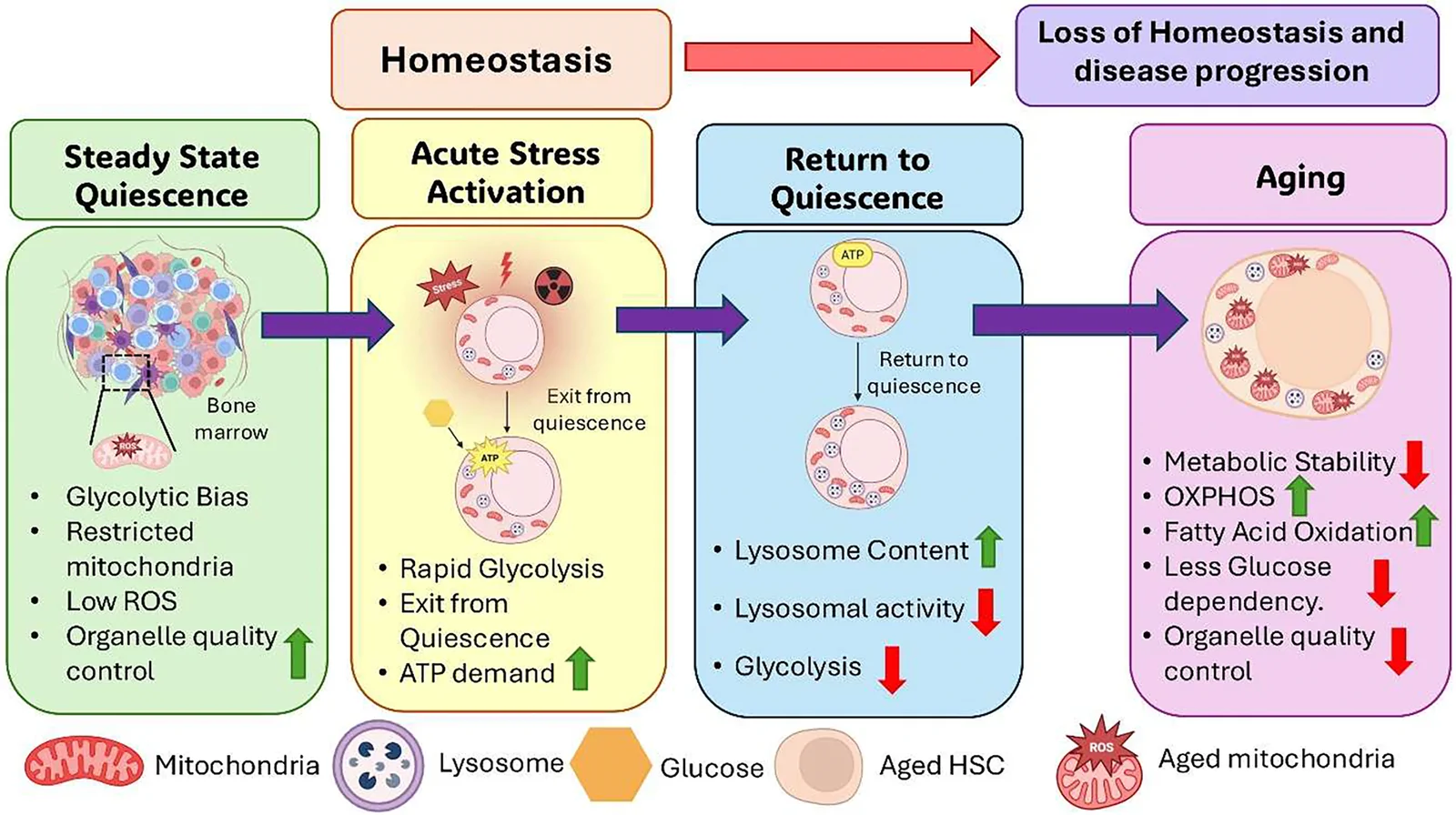
Hematopoietic stem cell (HSC) fate is shaped by dynamic metabolic control rather than a simple switch between glycolysis and oxidative phosphorylation (OXPHOS). At steady state, quiescent HSCs maintain low ROS and organelle quality control. During acute stress, HSCs exit quiescence and increase glycolysis to meet ATP demand. Recovery requires active metabolic reset and restoration of homeostasis. With aging, this balance becomes unstable and is associated with mitochondrial dysfunction, altered stress responses, and reduced niche support.

Once HSCs undergo stress driven proliferation, they need to cut down the activated state in a controlled way to avoid exhaustion. Work from Baumgartner et al. showed that an ERK-dependent feedback mechanism helps regulate HSC fitness during emergency hematopoiesis. It supports re-entry into quiescence by limiting excessive AKT/mTORC1 signaling [77]. This idea is also further supported by Pietras et al., who showed that reentry into quiescence protects HSCs from the damaging effects of chronic type I interferon exposure. This highlights that quiescence itself acts as a safeguard during recovery from stress [78]. From a metabolic standpoint, return to quiescence requires moving away from the glycolysis-ready primed state and returning to restrained organelle homeostasis. Mitochondrial support and quality control seem to be important for HSC recovery from stress. One study suggests that peroxisome proliferator-activated-γ coactivator-1α (PGC-1α) is important for hematopoietic recovery under stress conditions [79]. PGC-1α is a major regulator of mitochondrial biogenesis in HSCs. It, however, has less effect under basal conditions pointing to the importance of mitochondrial capacity when the system is pushed beyond steady-state demand. Another work also showed that FOXO3A initiates a protective autophagy program after ex vivo cytokine withdrawal and in vivo calorie restriction in HSCs. It also helps them tolerate metabolic stress and alleviate energy crisis, allowing their survival [48]. Mitochondrial clearance through mitophagy is likely needed [56,80], although it has not yet been demonstrated directly. A recent study shows that nuclear receptor coactivator 2, *Ncoa2*, promotes HSC return to quiescence after BM irradiation through *Foxo3a*-dependent mitophagy [81]. Together, these studies suggest that during acute stress hematopoiesis, mitochondrial biogenesis, mitophagy, and autophagy help sustain recovery and protect HSC function under continued stress (Figure 2 and Table S1).

Yet, return to quiescence is not sufficient to fully maintain HSC functions. HSC functions decline with division history [43,82–84]. Loss of mitochondrial quality control through cumulative division and subsequent metabolic drifts likely contribute the aging phenotype. We recently showed that HSC that have history of division no longer catabolize BCAA into intermediate metabolite, instead the BCAA leucine remains abundant and promotes mTOR activation for nucleotide synthesis; as a result, cell cycling is accelerated [43].

## BONE MARROW NICHE SIGNALS AND HSC METABOLIC AGING

Age related HSC changes are not only cell intrinsic but are also a result of changes in the bone marrow microenvironment. Under steady state conditions, the niche controls oxygen availability, nutrient access, and local signals that influence whether HSCs remain quiescent or activated. Key niche factors such as CXCL12 and SCF/KITL help support HSC retention in the bone marrow in a low activation state [85]. During stress, this support becomes altered. Niche inputs shift and in aging, niche quality declines which can push chronic stress-like signaling [86–88]. Aged HSCs respond differently to cytokines [89]. Young et al. (2021) [61] showed that hallmarks of HSC aging begin to appear during middle age due to reduced local insulin-like growth factor 1 IGF-1 in the bone marrow niche. Reduced IGF-1 input is one cause of HSC aging phenotype and reduced mitochondrial functions that can be restored by direct IGF-1 stimulation. Chronic inflammation alters aged HSC metabolic activity [51]. Other components also decline. Vascular niche-forming vessels decline and stromal and endosteal inflammatory signaling increases, which can keep HSCs in a continuous stress state preventing them to return to steady state [86,88]. Repeated niche cues can push metabolic circuits that are meant to be transient. Accumulative division is also a cause of aging [83].

## AGING AND FAILURE OF MITOCHONDRIAL STRESS MANAGEMENT CHECKPOINTS: A THERAPEUTIC OPPORTUNITY

NAD^+^ is a central metabolic cofactor, and the NAD^+^-dependent checkpoint is a system that links cellular NAD^+^ status to mitochondrial activation, stress handling, and HSC fate during proliferation [90]. In young HSCs, this checkpoint helps support activation in a controlled way by allowing mitochondrial metabolism to increase when needed. Studies have shown that CD38 promotes HSC proliferation in young mice by driving mitochondrial Ca^2+^ influx and mitochondrial metabolism. However, abnormal CD38 upregulation during aging disrupts NAD^+^ metabolism and compromises mitochondrial stress management [87]. As a result, mitochondrial activation in aged HSCs is no longer mediated by proper stress control, which can contribute to loss of functional stability. Another study has shown that that *Sirt3* expression is suppressed in aged HSCs and restoring *Sirt3* improved their regenerative capacity. SIRT3 is a mammalian sirtuin that is abundant in HSCs where it regulates a stress response. It mediates the global acetylation landscape of mitochondrial proteins and reduces oxidative stress [91]. This idea is also supported by studies using NAD^+^ boosting strategies. Treatment with nicotinamide riboside was shown to improve hematopoiesis through increased mitochondrial clearance. In aged HSCs, NAD^+^ restores a more youthful metabolic state by reducing mitochondrial stress, mitochondrial mass, and mitochondrial network size [64,80]. Consistently, urothilin A, a gut metabolite that stimulates mitophagy, can reverse HSC aging phenotype with increased lymphoid potential and the regenerative potential of aged HSCs, subsequently improving immune responses to viral infection in aged mice [92]. Finally, pharmacologic activation of chaperone-mediated autophagy reduces intracellular ROS, increases glycolysis, and restores long-term engraftment potential of aged HSCs [74]. Together, these findings suggest that aging is not simply associated with more mitochondrial activity, but with poorly controlled mitochondrial activation and weaker stress-management checkpoints (Figure 2 and Table S1).

## CONCLUSIONS

HSC functions across steady state, acute stress, aging, and disease cannot be defined as a simple switch between glycolysis and oxidative phosphorylation. During steady state, HSCs remain quiescent through a coordinated metabolic checkpoint that includes glycolytic bias, restricted mitochondrial activity, low ROS, and active organelle quality control. This is also closely associated with the bone marrow niche, where local factors, vascular signals, and oxygen levels help maintain HSC support and retention. During acute stress hematopoiesis, this balance temporarily shifts to meet the sudden regenerative demand. Early activation appears to depend strongly on rapid glycolytic adaptation; however, sustained recovery requires mitochondrial support, mitochondrial quality control, and metabolic flexibility. More importantly, return to quiescence is an active reset in which proliferative signaling must be turned down and mitochondrial and lysosomal homeostasis should be restored. With aging, this control becomes less stable. Instead of a simple increase in OXPHOS, aged HSCs show a gradual drift in mitochondrial homeostasis. This is assisted by altered stress management, and reduced ability to maintain the tightly controlled balance seen in young HSCs. At the same time, ageassociated changes in the bone marrow niche further weaken HSC support. This might create conditions that favor the expansion of mutant clones with stronger metabolic fitness. This idea became clearer in clonal hematopoiesis, as mutant stem and progenitor cells, and later leukemia stem cells in leukemic patients, were seen to benefit through stronger mitochondrial fitness and metabolic plasticity. This is an important area of research that needs further investigation.

## Supplementary Material

Table S1

The following supplementary materials are available online, Table S1: Summary of the main metabolic pathways regulating HSC functions.

## Data Availability

No data were generated from the study.
