# Supplementary material for: Hematopoietic Stem Cell Metabolism during Stress and Aging
Source: Adv Geriatr Med Res. Author manuscript; Available in PMC 2026 Aug 29. (PMC13521646; doi:10.20900/agmr20260018)
Supplement: Supplementary file 1 [file NIHMS2198377-supplement-Table_S1.pdf]

Supplementary Material

Table S1. Summary of the main metabolic pathways regulating HSC functions.

Pathway	Young or steady-state HSCs	Stress or aged HSCs	Key regulators/mechanisms	Citations
Gycolysis	Supports quiescence and limits mitochondrial dependence	Stress increases glycolytic demand. Aging reduces metabolic flexibility	HIF-1α, MEIS1, PDK, PFKFB3	[1,2]
Oxidative phosphorylation	Restrained but still required for HSC maintenance	Increased or dysregulated with aging; causes mitochondrial stress and altered resilience	Mitochondrial ATP, SDHAF1, SIRT7/UPRmt	[3–5]
Fatty acid oxidation/lipid metabolism	Supports HSC maintenance, self-renewal, and asymmetric division	Altered lipid handling may affect mitochondrial fitness and stress adaptation	PML-PPARδ, CPT1, CD36	[6–8]
BCAA/amino acid metabolism	Helps maintain amino acid balance and HSC fitness	Dysregulated amino acid availability or catabolism may impair replication-dependent HSC function	BCAA catabolism	[9]
Pentose phosphate pathway/NADPH balance	Supports redox balance and cell growth	Important during oxidative or regenerative stress.	G6PD, NADPH	[5,10,11]
NAD^+ metabolism	Supports mitochondrial function, DNA repair, and stress resistance	NAD^+ decline or CD38-driven NAD^+ consumption contributes to aged HSC dysfunction	NAD^+, CD38, SIRT proteins, PARPs	[12,13]
Autophagy/mitophagy	Maintains quiescence and metabolic quality control by clearing mitochondria	Declines or becomes insufficient with aging, allowing mitochondrial/metabolic stress to accumulate	ATG pathways, mitochondrial clearance, SIRT3	[14–16]
Lysosomal function/CMA	Supports protein quality control and HSC activation capacity	Lysosomal and CMA dysfunction contribute to aged HSC defects	LAMP2A, HSC70, lysosomal activity, CMA activation	[17–19]
Mitochondrial ROS control	Low ROS supports quiescence and long-term HSC potential	Increased ROS promotes DNA damage, differentiation bias, and functional decline	FOXO proteins, SIRT3, mitochondrial homeostasis	[16,20]
IGF1 and niche-derived growth signals	Supports balanced regenerative and mitochondrial responses	Age-associated IGF1 decline in the bone marrow niche contributes to HSC aging	IGF1, niche-derived metabolic support	[21]

REFERENCES

1. Simsek T, Kocabas F, Zheng J, Deberardinis RJ, Mahmoud AI, Olson EN, et al. The distinct metabolic profile of hematopoietic stem cells reflects their location in a hypoxic niche. Cell Stem Cell. 2010;7(3):380–90.
2. Takubo K, Nagamatsu G, Kobayashi CI, Nakamura-Ishizu A, Kobayashi H, Ikeda E, et al. Regulation of glycolysis by Pdk functions as a metabolic checkpoint for cell cycle quiescence in hematopoietic stem cells. Cell Stem Cell. 2013;12(1):49–61.
3. Anso E, Weinberg SE, Diebold LP, Thompson BJ, Malinge S, Schumacker PT, et al. The mitochondrial respiratory chain is essential for haematopoietic stem cell function. Nat Cell Biol. 2017;19(6):614–25.
4. Mohrin M, Shin J, Liu Y, Brown K, Luo H, Xi Y, Haynes CM, et al. Stem cell aging. A mitochondrial UPR-mediated metabolic checkpoint regulates hematopoietic stem cell aging. Science. 2015;347(6228):1374–7.
5. Watanuki S, Kobayashi H, Sugiura Y, Yamamoto M, Karigane D, Shiroshita K, et al. SDHAF1 confers metabolic resilience to aging hematopoietic stem cells by promoting mitochondrial ATP production. Cell Stem Cell. 2024;31(8):1145–61 e15.
6. Ito K, Carracedo A, Weiss D, Arai F, Ala U, Avigan DE, et al. A PML-PPAR-delta pathway for fatty acid oxidation regulates hematopoietic stem cell maintenance. Nat Med. 2012;18(9):1350–8.

7. Mistry JJ, Hellmich C, Moore JA, Jibril A, Macaulay I, Moreno-Gonzalez M, et al. Free fatty-acid transport via CD36 drives beta-oxidation-mediated hematopoietic stem cell response to infection. Nat Commun. 2021;12(1):7130.
8. Merchant S, Paul A, Reyes A, Cassidy D, Leach A, Kim D, et al. Different effects of fatty acid oxidation on hematopoietic stem cells based on age and diet. Cell Stem Cell. 2025;32(2):263–75 e5.
9. Bartram J, Treichel S, Song BA, Xu J, Sharma D, Nasr W, et al. Activation of a branched-chain amino acid rheostat restores replication-dependent hematopoietic stem cell fitness. Cell Stem Cell. 2026;33(2):289–305 e6.
10. Morganti C, Bonora M, Ito K. Metabolism and HSC fate: what NADPH is made for. Trends Cell Biol. 2025;35(10):866–79.
11. Tao S, Su M, Yu C, Qiu X, Zou B, Qiu R, et al. Post-irradiation dietary restriction impairs hematopoiesis via inhibition of the pentose phosphate pathway in hematopoietic stem and progenitor cells. Cell Death Dis. 2026;17(1):8.
12. Sun X, Cao B, Naval-Sanchez M, Pham T, Sun YBY, Williams B, et al. Nicotinamide riboside attenuates age-associated metabolic and functional changes in hematopoietic stem cells. Nat Commun. 2021;12(1):2665.
13. Song Z, Park SH, Mu WC, Feng Y, Wang CL, Wang Y, et al. An NAD(+)-dependent metabolic checkpoint regulates hematopoietic stem cell activation and aging. Nat Aging. 2024;4(10):1384–93.
14. Ho TT, Warr MR, Adelman ER, Lansinger OM, Flach J, Verovskaya EV, et al. Autophagy maintains the metabolism and function of young and old stem cells. Nature. 2017;543(7644):205–10.
15. Fang Y, An N, Zhu L, Gu Y, Qian J, Jiang G, et al. Autophagy-Sirt3 axis decelerates hematopoietic aging. Aging Cell. 2020;19(10):e13232.
16. Brown K, Xie S, Qiu X, Mohrin M, Shin J, Liu Y, et al. SIRT3 reverses aging-associated degeneration. Cell Rep. 2013;3(2):319–27.
17. Dong S, Wang Q, Kao YR, Diaz A, Tasset I, Kaushik S, et al. Chaperone-mediated autophagy sustains haematopoietic stem-cell function. Nature. 2021;591(7848):117–23.
18. Liang R, Arif T, Kalmykova S, Kasianov A, Lin M, Menon V, et al. Restraining Lysosomal Activity Preserves Hematopoietic Stem Cell Quiescence and Potency. Cell Stem Cell. 2020;26(3):359–76 e7.
19. Arif T, Qiu J, Khademian H, Lohithakshan A, Menon A, Menon V, et al. Reversing lysosomal dysfunction restores youthful state in aged hematopoietic stem cells. Cell Stem Cell. 2025;32(12):1904–22 e7.
20. Morganti C, Ito K. Mitochondrial Contributions to Hematopoietic Stem Cell Aging. Int J Mol Sci. 2021;22(20).
21. Young K, Eudy E, Bell R, Loberg MA, Stearns T, Sharma D, et al. Decline in IGF1 in the bone marrow microenvironment initiates hematopoietic stem cell aging. Cell Stem Cell. 2021;28(8):1473–82 e7.
